# Renal glomus tumor: A case report and literature review

**DOI:** 10.1016/j.eucr.2024.102813

**Published:** 2024-07-26

**Authors:** Chi-Chun Hsieh, Yung-Shun Juan, Yi-Ting Chen

**Affiliations:** a Department of Urology, Kaohsiung Medical University Chung-Ho Memorial Hospital, No.100, Tzyou 1st Road, Kaohsiung, 807, Taiwan; b Department of Pathology, Kaohsiung Medical University Chung-Ho Memorial Hospital, No.100, Tzyou 1st Road, Kaohsiung, 807, Taiwan

**Keywords:** Glomus tumor, Partial nephrectomy, Renal tumor, Urinary tract disease

## Abstract

Glomus tumors are rare mesenchymal tumors involving cells from the glomus body, smooth muscle, and vasculature, typically found in distal extremities' skin. This case describes a 54-year-old woman with a history of hypothyroidism and hyperlipidemia, incidentally discovered to have a four-centimeter calcified renal tumor. Surgery was performed due to suspected malignancy. Immunohistochemical staining confirmed a renal glomus tumor, positive for muscle actin and smooth muscle actin (SMA). The tumor was benign, and no adjuvant therapy was needed. The patient remained recurrence-free during follow-up. Renal glomus tumors are predominantly benign, with surgical resection as the primary treatment.

## 1 Introduction

Glomus tumors, resembling the glomus body, are uncommon perivascular neoplasms. These rare soft tissue tumors exhibit similar incidences in both sexes, predominantly affecting individuals aged twenty to forty years.^1^ Various variants of glomus tumors exist, including glomangioma, glomangiomyoma, and glomangiomatosis. While commonly found in the skin of distal extremities, such as the subungual area of fingers, palms, wrists, forearms, and feet, their occurrence in visceral organs like the mediastinum, lung, gastrointestinal tract and kidney is infrequent.^2^ Other rare renal tumors include lymphoma and leiomyoma.^3^^,^^4^ Currently, there are no definitive guidelines for the treatment of renal glomus tumors. This report presents a case of a 54-year-old woman diagnosed as primary renal glomus tumor, accompanied by review of literature.

## 2 Case Presentation

**Fig. 1:**
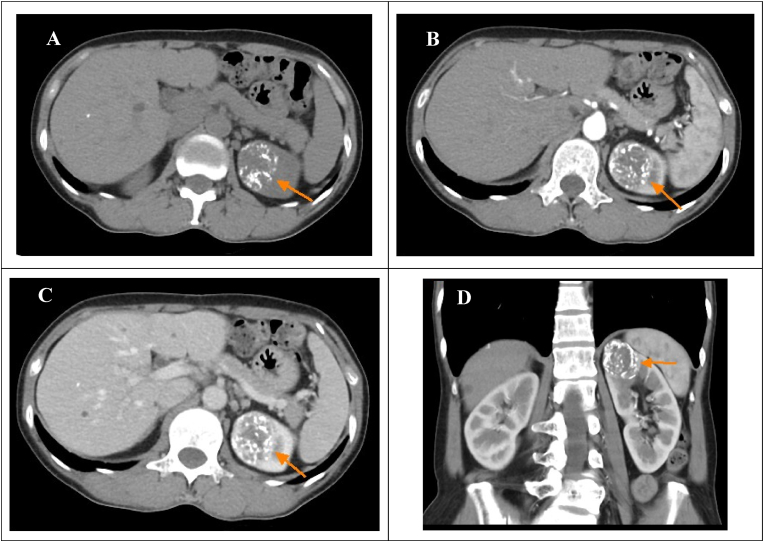
CT imaging of our cases (A) Precontrast CT showed a heterogenous renal tumor with calcification over the upper pole (B) Arterial phase (C) Venous phase (D) Coronal view.

A 54-year-old woman with a medical history of hypothyroidism and hyperlipidemia presented with a left renal tumor approximately 4 cm in diameter, incidentally discovered on computed tomography (CT) (Fig. 1). The tumor displayed calcifications and septa in the upper pole, with partial enhancement and heterogeneity, and was bordered clearly. While the patient reported no hematuria or palpable mass, occasional left flank pain was noted.

**Fig. 2:**
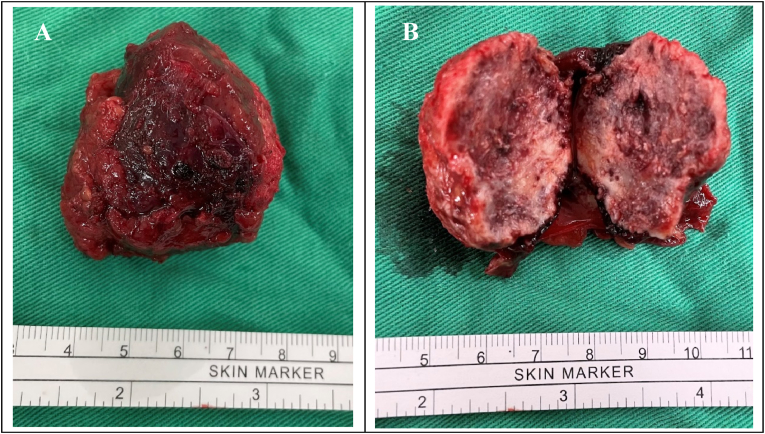
Tumor specimen (A) Gross tumor specimen (B) Cross section of the tumor.

Following thorough discussion, she elected for laparoscopic partial nephrectomy due to suspected renal cell carcinoma. The surgical procedure proceeded smoothly without complications, resulting in the resection of a tumor measuring 4.5 × 3x3 cm confined to the renal parenchyma (Fig. 2).

**Fig. 3:**
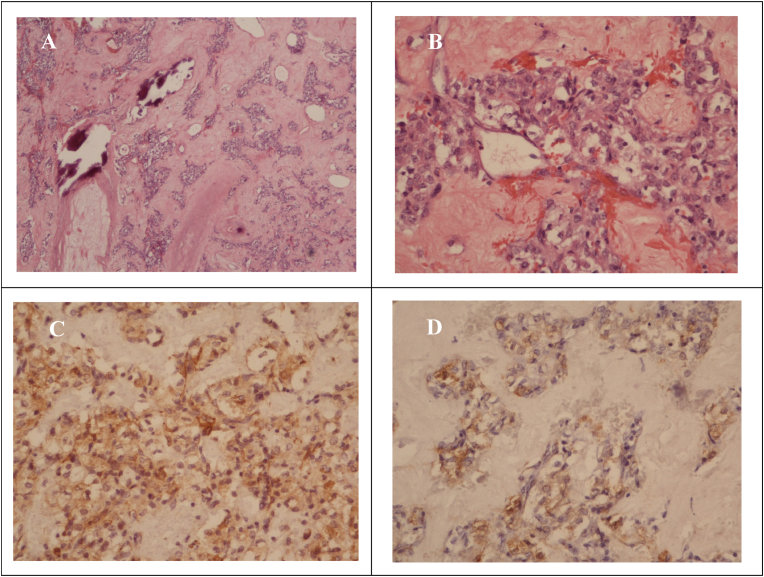
Immunochemical staining of tumor specimens Immunochemical staining of tumor specimen (A) Hematoxylin and eosin (H&E) staining 40X (B) H&E) staining 200X (C) Immunoactivity for muscle actin (D) Immunoactivity for smooth muscle actin.

Histopathological examination, coupled with immunohistochemical staining, disclosed immunoreactivity for muscle actin and smooth muscle actin (SMA), confirming the diagnosis of a glomus tumor (Fig. 3). No expression of Desmin, CD31, CD34, CK, Synaptophysin, PAX-8, HMB-45, INSM-1, or GLUT-1 was observed within the tumor cells. Additionally, microscopic examination indicated chronic pyelonephritis in the excised portion. The patient was discharged on the sixth post-operative day, with subsequent follow-up revealing no impairment in renal function or tumor recurrence.

## 3 Discussion

The glomus body serves as a specialized anastomosis between artery and vein primarily regulating heat, commonly found in the extremities, including digits and palms, residing within the stratum reticularis of the dermis.^5^

Glomus tumors, mesenchymal tumors resembling the normal glomus body, composed of vasculature and smooth muscle, were first proposed by Masson in 1924. He described three cases presenting with intermittent sharp pain in the upper extremity, which alleviated post-tumor excision, noting structural similarities to the glomus body and suggesting a relation to hyperplasia or overgrowth of the glomus body.

The estimated incidence of glomus tumors, as reported by the Mayo Clinic, stands at 1.6 % among 500 consecutive soft tissue tumors. Both sexes exhibit an similar incidence, with subungual lesions being more common in females. Glomus tumors typically afflict individuals aged between twenty and forty, often with symptoms preceding diagnosis.

While the subungual region represents the most frequent site for glomus tumors, occurrences in other extremities such as the foot, forearm, palm, and wrist are also noted. Rare instances of glomus tumors have been reported in various locations, including the genital organs, oral cavity, trachea, mediastinum, heart, and lymph nodes. Although typically solitary, reports exist of multiple glomus tumors at subungual region in patients with neurofibromatosis 1 (NF1). Symptoms often do not correlate with tumor size; some individuals experience radiating pain triggered by changes in temperature or minor tactile stimulation. We listed cases of reported renal glomus tumor in Table 1.^2^^,^^6^, ^7^, ^8^, ^9^, ^10^, ^11^, ^12^, ^13^, ^14^, ^15^

**Table 1:** Review of previously reported renal glomus tumors.

Reference	Age, Sex	Size (cm)	Location of kidney	Image findings	Treatment	Positive immunochemical stain	Follow-up
**Benign Glomus Tumor**							
Siddiqui et al., 2005^9^	55, Female	2	Left lower pole	Not stated	Partial	SMA, vimentin	Not stated
Heraw et al., 2005^8^	53, Female	2.5	Right ureteropelvic junction	CT: Solid mass with hydronephrosis Nonfunctioning kidney and atrophy of the surrounding parenchyma	Radical	SMA, collagen type 4	Free of disease at 6 months
Al-Ahmadie et al., 2007^10^	36, Male	3.3	Right anterior interpole	Sonography: Peripherally echogenic and centrally hypoechoic, conﬁned to the renal capsule	Partial	SMA, muscle actin	Free of disease at 62 months
Al-Ahmadie et al., 2007	81, Male	4	Right lower pole	Not stated	Radical	SMA	Free of disease at 24 months
Al-Ahmadie et al., 2007	48, Male	7.3	Right mid/lower pole	CT: Moderately enhancing, multilobulated	Radical	SMA	Free of disease at 33 months
Sasaki et al., 2011^2^	62, Male	1.8	Left lower pole	CT: Enhanced lesion	Partial	SMA, vimentin, CD57, collagen type 4	Free of disease at 2 months
Gravet et al., 2015^13^	60, Male	2.5	Left upper pole	CT: Enhanced, exophytic lesion	Partial	SMA, vimentin	Free of disease at 8 months
Present report	54, Female	4	Left upper pole	CT: calcifications and septa	Partial	SMA, muscle actin	Free of disease at 16 months
**Atypical and Malignant Glomus Tumor**							
Gill and Van Vliet, 2010^11^	46, Male	8.7	Right lower pole	CT: Exophytic, irregular peripheral enhancement, with septations, and central necrosis	Radical	SMA, MSA, CD34, bcl-2, vimentin, synaptophysin	Free of disease at 15 months
Lamba et al., 2011^20^	44, Male	Metastasis primary tumor size unknown	Posterior right kidney Metastasis to spine and pelvic bones	CT: Mixed cystic and solid components arising from the posterior right kidne Multiple osseous metastases involving the spine and pelvic bones	Palliative RT + C/T	SMA, CD34, vimentin, collagen type 4	Died of disease at 6 months
Lai et al., 2016 and Chen et al., 2017^14^^,^^15^	46, Male	3.7	Right upper pole	Sonography: Slightly hyperechoic CT: Contrast-enhanced, heterogeneous	Radical	Not stated	Free of disease at 6 months
Li et al., 2018^6^	31, Female	16	Right	CT: Heterogeneous mass with an area of central necrosis	Radical	Vimentin, collagen type 4	Recurrence after 7 years of follow-up Died of disease at 13 years
Li et al., 2018	33, Female	9.7	Left with renal vein and IVC thrombus and tricuspid valve vegetaion	Not stated	Radical	Vimentin, MSA	Not stated
Li et al., 2018	55, Male	1.5	Left	Sonography: cystic mass	Partial	SMA, collagen type 4	Not stated
Zhao et al., 2020^7^	8, Female	5	Right upper pole	CT: Well-demarcated, hypodense, solid, with homogeneous contrast enhancement	Partial	SMA, MSA, vimentin, collagen type 4, CD34, renin	Free of disease at 16 months

Note: C/T, chemotherapy; CT, computed tomography; IVC, inferior vena cava, MSA, muscle specific actin; RT, radiotherapy; SMA, smooth muscle actin.

Primarily benign, renal glomus tumors occasionally manifest malignantly.^8^^,^^10^^,^^13^ Reported symptoms, including abdominal, flank discomfort or microscopic hematuria, are nonspecific, with most cases diagnosed incidentally under imaging.

Diagnosing a glomus tumor of the kidney solely based on imaging remains challenging due to its low incidence rate and radiologic characteristics.^16^ While radiological imaging may reveal enhanced, heterogeneous lesions with clear borders on CT scans, tissue biopsy or pathology from surgery is essential for confirmation. Immunohistochemical analysis aids in differentiation from renal cell carcinoma, with positive immunoreactivity for muscle markers and negative expression of epithelial markers distinguishing renal glomus tumors.

A previous study established criteria for potential malignancy in renal glomus tumors, including tumor size exceeding 2 cm and deep location within the kidney, the presence of atypical mitotic figures, and prominent nuclear grade and mitotic activity (5 mitoses/50 High-power field).^17^ In our case, the tumor exceeded 2 cm in size and was situated deep in the left upper pole of kidney, with the pathology report indicating no evident increased mitosis or necrosis. Surgical resection remained the primary treatment modality in previously documented cases. Overall, the prognosis was deemed acceptable, with pathological reports indicating either atypical or malignant features or benign glomus tumors. Notably, Lamba et al. reported the first instance of malignant glomus tumors of kidney with pelvic bone and spine metastasis, despite receiving palliative radiation therapy or chemotherapy, resulting in an undesirable response and eventual demise within months of diagnosis.^12^ As far as we know, only one malignant glomus tumor of kidney has been documented in our area according to these criteria. Following the tumor diagnosis, the patient underwent radical nephrectomy with adrenalectomy, and he remained free of disease at the six-month follow-up.

Currently, nephrometry scoring systems like the RENAL score prove useful in evaluating functional outcomes post-partial nephrectomy in renal tumors.^18^^,^^19^

## 4 Conclusion

In conclusion, renal glomus tumors present as uncommon neoplasms with nonspecific clinical symptoms, posing challenges in differentiation diagnosis between other renal malignancy based on clinical manifestation, laboratory data, and clinical imaging. Definitive diagnosis relies on histopathological examination. Although treatment consensus remains scarce due to their rarity, considering the favorable outcomes and low recurrence rates, surgical resection, whether partial or radical nephrectomy, remains a viable option. Regular post-treatment follow-up may be warranted due to reported instances of recurrence, albeit infrequent.

## Declaration of interest

There were no potential financial or nonfinancial conflicts of interest.

## Ethics approval and consent to participate

This study was approved by the Institutional Review Board of Kaohsiung Medical University Chung-Ho Memorial Hospital (KMUHIRB-E(I)-20230,151).

## Consent for publication

Not applicable.

## Availability of data and materials

The datasets generated and/or analyzed during the current study are available in the PubMed repository, https://pubmed.ncbi.nlm.nih.gov/

## Competing interests

The authors declare that they have no competing interests.

## Fundings

Not applicable.

## CRediT authorship contribution statement

**Chi-Chun Hsieh:** Conceptualization, Writing – original draft. **Yung-Shun Juan:** Supervision. **Yi-Ting Chen:** Data curation.
